# Evaluation of three scales of dyspnea in chronic obstructive pulmonary disease

**DOI:** 10.4103/1817-1737.53351

**Published:** 2009

**Authors:** S. K. Chhabra, A. K. Gupta, M. Z. Khuma

**Affiliations:** *Department of Cardiorespiratory Physiology, Clinical Research Centre, Vallabhbhai Patel Chest Institute, University of Delhi, Delhi, India*

**Keywords:** Baseline dyspnoea index, chronic obstructive pulmonary disease, Modified Medical Research Council scale, oxygen cost diagram

## Abstract

**BACKGROUND:**

The Modified Medical Research Council (MMRC) scale, baseline dyspnea index (BDI) and the oxygen cost diagram (OCD) are widely used tools for evaluation of limitation of activities due to dyspnea in patients with chronic obstructive pulmonary disease (COPD). There is, however, limited information on how these relate with each other and with multiple parameters of physiological impairment.

**OBJECTIVES:**

To study the interrelationships between MMRC, BDI and OCD scales of dyspnea and their correlation with multiple measures of physiological impairment.

**MATERIALS AND METHODS:**

A retrospective analysis of pooled data of 88 male patients with COPD (GOLD stages II, III and IV) was carried out. Dyspnea was evaluated using the MMRC, BDI and OCD scales. Physiological impairment was assessed by spirometry (FVC % predicted and FEV_1_ % predicted), arterial blood gas (ABG) analysis and measurement of the 6-min walk distance (6MWD).

**RESULTS:**

The interrelationships between MMRC, BDI and OCD scales were moderately strong. The BDI and OCD scores had strong correlations with ABG abnormalities, weak correlations with spirometric parameters but none with 6MWD. MMRC grades were significantly associated with BDI and OCD scores but did not show clear associations with spirometric parameters, ABG abnormalities and 6MWD.

**CONCLUSIONS:**

The MMRC grades of dyspnea and the BDI and OCD scales are moderately interrelated. While the BDI and OCD scales have significant associations with parameters of physiological impairment, the MMRC scale does not.

The American Thoracic Society (ATS) has defined breathlessness, or dyspnea, as a “subjective experience of breathing discomfort that is comprised of qualitatively distinct sensations that vary in intensity.”[1] It is a complex symptom of cardiopulmonary diseases, including chronic obstructive pulmonary disease (COPD), and affects several dimensions of a patient's life, reducing activity and functional capacity besides causing substantial distress and discomfort. The GOLD (Global Initiative for Chronic Obstructive Lung Disease) guidelines for grading the severity of COPD have recommended the use of a staging system based on post-bronchodilator FEV_1_.[2] Physiological assessments, however, provide only limited information about the impact of disease, and therefore a multidimensional approach has been recommended for evaluation. [3,4] Assessment of dyspnea is critical in patient evaluation and management as it is the major limiting factor for activities of daily living and is also a superior predictor of mortality than spirometry.[5] It is included in the body mass index, airflow obstruction, dyspnea, and exercise capacity (BODE) index, a multidimensional scoring system that predicts mortality[6] and risk of hospitalization.[7]

Among the more commonly used validated scales to evaluate dyspnea in COPD[8] are the Modified Medical Research Council (MMRC) [9] and the closely related ATS78 scale,[10] the baseline dyspnea index (BDI),[11] and the oxygen cost diagram (OCD).[12] Although differing widely in content and scoring, these scales were found to be significantly interrelated.[13] Studies using the technique of factor analysis have also found that these scales club in the same domain.[14,15] Pulmonary function tests, arterial blood gas analysis and measurement of exercise capacity such as the 6-min walk distance (6MWD) are commonly used clinical tools to evaluate the physiological and functional impairment caused by COPD. In separate evaluations, these scales of dyspnea have been found to have weak or no correlation with lung function parameters.[13–15] As these scales have wide differences in content, their construct validity against physiological parameters is likely to differ. We therefore studied the relationship of these scales with multiple parameters of physiological impairment.

## Materials and Methods

We carried out a retrospective analysis of pooled data of two recent studies on COPD[16,17] done in our Institute. These had common outcomes, including dyspnea, spirometry, arterial blood gases and 6MWD measured with standardized methodology. The studies were approved by the Institutional Ethics Committee. Informed consent was taken.

Eighty-eight ambulatory stable male patients that were all former or current smokers with at least 10 pack-years of smoking and diagnosed with moderate or severe COPD (stages II, III and IV) on the basis of GOLD recommendations[2] were investigated. Only males were included as smoking-induced COPD is uncommon in the female population in India due to their substantially lower habit of smoking. The number of female patients reporting to our practice is therefore limited. Patients were excluded if there was a history suggestive of asthma, an acute exacerbation of COPD in the previous 4 weeks, and evidence of any other concurrent respiratory disorder or any systemic disease such as ischemic heart disease, musculoskeletal disorders, peripheral vascular diseases or other disabling conditions that would interfere with the tests.

## Study design

The study was carried out in the outpatient setting between 10 a.m. and 12 noon. Bronchodilators were withdrawn 12 hours before the investigation. Inhaled corticosteroids were however allowed unchanged. After a detailed history and examination, the patients had an evaluation of dyspnea using the three instruments in a random order, followed by an arterial blood gas analysis and the 6-min walk test. After the patient had rested for some time, spirometry before and after administration of bronchodilator was carried out.

## Methods

Dyspnea assessment was carried out using the MMRC, BDI and the OCD scales. The MMRC is an ordinal five-point scale (grades I to V) based on degrees of various physical activities that precipitate dyspnea.[9] Grade V represents the most severe category. The BDI has a scale of five grades, scored 0 to 4, for each of the following categories: Functional impairment, magnitude of task and magnitude of effort.[11] The ratings for each of the three categories were added to give the total score (range, 0-12). Lower the score, greater is the dyspnea. The OCD is a visual analog scale that corresponds to oxygen requirements at different activity levels, which are represented as a value ranging from zero to 100mm with the highest score indicating no impairment.[12] The patient is asked to indicate the level of activity at which he/she begins to experience dyspnea. The OCD score is measured in 100mm. The shorter the distance, the greater is the breathlessness.

The 6-min walk test was carried out as recommended by the American Thoracic Society.[18] The patients were instructed to walk in a corridor on level at their own pace attempting to cover as much ground as possible in 6 minutes. They were constantly encouraged using standard phrases to put in their maximal effort but were permitted to slow down or stop and restart during the test. The distance traveled within 6 minutes (6MWD) was noted. Supplemental oxygen was not used during the test. The test was carried out only if oxygen saturation on pulse oximetry was greater than 88%. Only 1 patient was excluded by this criterion.

Spirometry was performed on the dry, rolling-seal spirometer of the Benchmark model lung function machine (P.K. Morgan, Kent, UK) as per the recommendations of the ATS.[19] Three acceptable and at least two reproducible maximal expiratory flow volume curves were obtained. The highest values of FEV_1_ (forced expiratory volume in 1 s) and FVC (forced vital capacity) obtained were recorded. Post-bronchodilator values were obtained by repeating the spirometry 20 minutes after inhalation of 200 μg salbutamol from a metered-dose inhaler. Reference equations for north Indian adults were used to calculate the predicted values in terms of percentage.[20] Arterial blood gas (ABG) analysis was carried out on radial artery blood sample measured in instrumentation laboratory machine (model 1312). The ABG analysis was obtained after a 15-minute rest in supine position breathing room air.

## Statistical analysis

Statistical analysis was carried out with the help of SPSS 14.0 software for Windows and GraphPad Prism 4.02 for Windows. Multiple group comparisons of quantitative variables were made using analysis of variance (ANOVA), and inter-group comparisons were carried out using the Bonferroni's test to identify the significantly different groups. Coefficients of correlation for bivariate relationships were obtained using the Spearman rank correlation test. The relationship between the MMRC grade and BDI/OCD scores was evaluated by computing the polyserial correlation. This was done as the MMRC is an ordinal variable and Spearman's or Pearson's correlation analysis is not an appropriate test to evaluate relationships between ordinal and continuous variables like BDI or OCD scores. Polyserial correlation was computed using Lisrel 8.80 student edition software. Proportions of patients with different MMRC grades across GOLD stages were analyzed by chi-square test. A *P* value of <0.05 was considered statistically significant.

## Results

**Figure 1:**
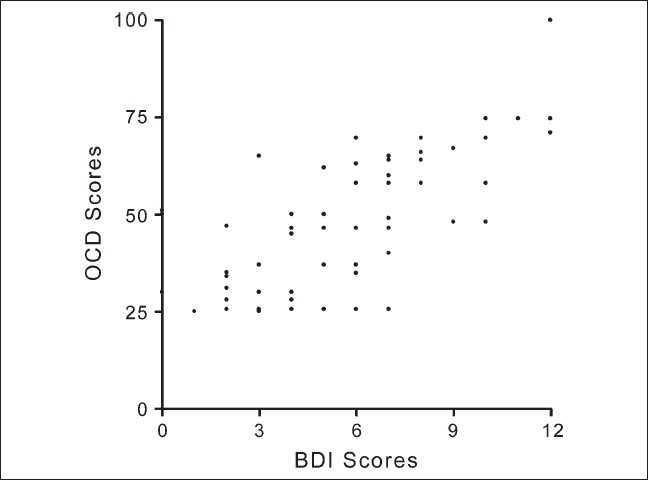
Correlation between BDI and OCD scores (r = 0.67, *P* <.0.1)

Table 1 shows the demographic and clinical characteristics according to GOLD stages. Table 2 shows the inter-correlations (spearman's rho) among spirometric, blood gas parameters, 6MWD, BDI and OCD. The OCD and BDI were moderately strongly correlated [Figure 1]. The correlations of these measures of dyspnea with FEV_1_ %predicted were weak although statistically significant, while there was no correlation with 6MWD. The correlations of BDI and OCD scores with arterial blood gas parameters were moderately strong. Polyserial correlation coefficients computed between MMRC grades and BDI and OCD were −0.56 (*P* <.05) and −0.54 (.05 > p <.1), respectively.

**Table 1:** Demographic and clinical characteristics according to GOLD stages

	GOLD II (n = 33)	GOLD III (n = 37)	GOLD IV (n = 18)
Age^ns^	58.5 (10.4)	58.6 (10.4)	57.0 (8.5)
Pack-years of smoking^ns^	32.4 (19.4)	41.1 (31.9)	45.8 (26.4)
Duration of disease^ns^	5.8 (4.4)	7.0 (6.0)	10.5.2 (8.9)
PaO_2_ (mm Hg)***	81.4 (7.0)	76.4 (9.8)	64.3 (9.3)+++@@
PaCO_2_ (mm Hg)**	40.2 (2.3)	43.7 (5.7)	47.0 (5.1)++
6-min walk	374.3 (128.6)	300.5 (120.5)^+^	235.9 (81.5)+++
distance (m)***			
MMRC grade**			
Grade II	16 (69.6)	4 (17.4)	3 (13)
Grade III	14 (33.3)	18 (42.9)	10 (23.8)
Grade IV	3 (13)	15 (65.2)	5 (21.7)
OCD score (mm)**	55.5 (16.6)	41.9 (15.6)++	44.0 (16.6)
BDI score***	6.9 (2.7)	4.5 (2.5)++	5.3 (2.2)
Post-BD FVC% predicted***	95.8 (13.1)	79.6 (12.0)+++	67.8 (14.6)+++@@
Post-BD FEV% predicted***	59.7 (7.2)	39.4 (5.4)+++	26.3 (5.2)+++@@@

All data represent mean (SD) except the MMRC grades that show frequency (row percentages). ANOVA results (column 1):

*** : *P* <.001,

** : *P* <.01, ns = not significant, *P* >.05; Post-hoc Bonferroni's test: *vs* column 2:

+++ : *P* <.001,

++ : *P* <.01; *vs* column 3:

@@@ : *P* <.001,

@@ : *P* <.01

**Table 2:** Correlation matrix of clinical severity variables

	PaO_2_ (mm Hg)	PaCO_2_ (mm Hg)	6-min walk distance (m)	OCD score	BDI score	Post-BD FVC% pred	Post-BD FEV_1_% pred
PaO_2_ (mm Hg)	1						
PaCO_2_(mm Hg)	−0.68**	1					
6-min walk distance (m)	0.29*	−0.27*	1				
OCD score	0.50**	−0.52**	0.20	1			
BDI score	0.50**	−0.52**	−0.02	0.67**	1		
Post-BD FVC % pred	0.33*	−0.34*	0.34**	0.5	0.08	1	
Post-BD FEV_1_ % pred	0.48**	−0.46**	0.41**	0.23*	0.22*	0.7**	1

Spearman's rho

* : *P* <.05;

** : *P* <.01

As the MMRC scale is an ordinal variable, we compared the different study variables across its different grades [Table 3]. The PaO_2_, PaCO_2_, post-bronchodilator FEV_1_ % predicted, OCD and BDI scores were significantly different between these categories, while the 6MWD and post-bronchodilator FVC % predicted were not. The PaO_2_, PaCO_2_ and post-bronchodilator FEV_1_ % predicted were significantly different between patients with MMRC grade 2 and 4, while grade 3 patients did not differ significantly for these variables with grade 2 and grade 4 patients. However, the OCD and BDI scores were significantly different for all the pairs of between-group comparisons, with scores indicating progressively increasing dyspnea with increasing MMRC grades.

**Table 3:** Study variables according to MMRC grade of dyspnea

	MMRC grade 2 (n = 23)	MMRC grade 3 (n = 42)	MMRC grade 4 (n = 23)
PaO_2_ (mm Hg)*	80.2 (7.8)	73.4 (13.1)	68.0 (6.9)+
PaCO_2_ (mm Hg)**	40.6 (2.2)	43.7 (5.5)	47.5 (5.7)++
6-min walk distance (m)^ns^	344.2 (145.3)	321.4 (121.6)	277.8 (112.6)
OCD score (mm)***	65.1 (11.9)	44.9 (12.6)+++	34.4 (14.6)+++@@
BDI score***	7.9 (2.1)	5.4 (2.5)+++	3.8 (2.0)+++@
Post-BD FVC % predicted^ns^	87.2 (15.9)	80.9 (16.5)	83.7 (17.7)
Post-BD FEV % predicted*	50.4 (14.9)	43.9 (13.7)	39.1 (12.8)+

All data represent mean (SD). ANOVA results (column 1):

*** : *P* <.001,

** : *P* <.01,

* : *P* <.05, ns = not significant, *P* >.05; Post-hoc Bonferroni's test: *vs* column 2:

+++ : *P* <.001,

++ : *P* <.01,

+ : *P* <.05; *vs* column 3:

@@ : *P* <.01,

@ : *P* <.05

## Discussion

The present study shows that the OCD and BDI scales of dyspnea measurement have a moderately strong correlation with each other in patients with COPD. Each of these scales had weak and moderately strong relationships with FEV_1_ % predicted and arterial blood gas abnormalities, respectively. The MMRC grade of dyspnea had a moderately strong correlation with BDI scores and somewhat weaker correlation with OCD scores. The BDI and OCD scores were significantly different between patients grouped according to MMRC grades. However, the categories of MMRC were not sufficiently discriminatory for spirometric and arterial blood gas abnormalities. None of the three measures of dyspnea was found to have a significant relationship with the 6MWD.

Previous studies have shown that the BDI and the OCD scores have significant although modest correlations with FEV_1_.[11] We observed weak correlations between these scales and FEV_1_ % predicted and none between these scales and FVC%predicted. In addition, these scales were also observed to have a moderately strong correlation with arterial blood gas abnormalities. This contrasts with the MMRC scale, which did not show any association with any of the measures of physiological impairment used in the present study. Bestall *et al.*[21] have also shown that FEV_1_ is not associated with grades of MMRC. The lack of relationship of MMRC grade with physiological parameters has also been shown in other studies, where it was however shown to be correlated more strongly with health-related quality of life and with indices of anxiety and depression than with spirometric values.[22,23] Hajiro *et al*[24] have shown that these three scales have similarly strong correlations with several indices of health-related quality of life. Thus, the BDI and the OCD scales appear to have a better construct validity. In a recent comparison of different scales by Eakin *et al,*[25] the BDI was found to have the highest levels of reliability and validity. Our results support this observation.

As these scales have been designed to capture the limitations imposed on daily activities by dyspnea, the significant inter-relationships among these are not surprising. In studies using factor analysis, these scales were found to group in the same factor, suggesting that these provide information about the same dimension of the disease.[14,15] Another study showed that the BDI scores correlated well with the MMRC grading and the OCD scores.[13]

Each of these scales has merits and limitations. In clinical practice, the MMRC grade remains the commonest scale used because of its simplicity, ease of administration and established validation as a useful marker in COPD. It predicts the likelihood of survival of patients with COPD,[5] correlates well with the health status scores[11,23,24] and is also a component of the BODE index.[6] Its limitations include a lack of significant association with any parameter of physiological and functional impairment. Further, it is primarily a discriminative instrument and is not very responsive, especially over short terms. Thus, its utility to evaluate response to treatment is limited. The BDI and the transition dyspnea index (TDI)[11] provide a method of assessing dyspnea at baseline (the BDI) and measuring subsequent changes over time following interventions (the TDI). It is more elaborate than the MMRC grade and is multidimensional. In addition to the magnitude of task required to induce dyspnea, it also rates functional impairment and the magnitude of effort needed to carry out a task. Thus, it is also more time consuming and complex. Being quantitative, these scales are amenable to mathematical operations. These have good discriminative and evaluative properties. The oxygen cost diagram (OCD) is a vertical scale designed to rate activities on a continuum according to the number of calories expended in the performance of the activity.[12] It is simple and easy to administer. However, not all patients engage in all the activities depicted along the scale, and some need repeated instructions for marking the appropriate response.[1] It is a unidimensional scale focusing on the magnitude of task.

The study has a few limitations. First, it is a retrospective analysis of pooled data from two studies. Although standardized methods were used, inter-observer differences may have influenced results. Second, only male patients were included for reasons explained earlier. This limits generality of results to female subjects. Gender differences in perception of dyspnea may modify the results.

## Conclusion

The inter-relationships among commonly used scales to measure dyspnea — the MMRC grading, BDI and OCD — are moderately strong in patients with COPD. However, the BDI and OCD scales are significantly associated with some of the measures of physiological impairment, while the MMRC grade is not.
